# Supplementary material for: Novel adomaviruses associated with blotchy bass syndrome in black basses (Micropterus spp.)
Source: PLoS One. 2025 Dec 17;20(12):e0326402. doi: 10.1371/journal.pone.0326402 (PMC12711042; doi:10.1371/journal.pone.0326402)
Supplement: S1 Fig — (PDF) [file pone.0326402.s001.pdf]

ANIMAL PATHOLOGY RECORD

DEPARTMENT OF ANIMAL PATHOLOGY
COLLEGE OF RESOURCE DEVELOPMENT
UNIVERSITY OF RHODE ISLAND
KINGSTON, R. I. 02881

Date Submitted: 11-20-84

Accession No.: 0 293

Date Received:

Veterinarian:

Address: 25 Main Street, Carolina, RI

Phone:

Owner:

Address:

Ident.	Animal	Breed	Color	Age	Sex	Weight	Prev. Acces.
Micropterus	salmoides				female		

Clinical Diagnosis:

History and Clinical Summary:
Fish collected by means of angling. Caught in Roundout Creek, tributary to the Hudson River Kingston, NY. A total of 81 bass were taken over an 8 hour period (7:30 a.m. - 3:30 p.m.). Dark external patches occurred only on fish over approx. 30 cm in length. The larger the specimmn, the more numerous the black patches. Not all fish over 30 cm exhibited these markings. They occurred on approx. 33% of fish between 30 cm and 35 cm. Specimens greater than 35 cm showed these markings approx. 50%+ of the time. The number of patches varied from fish to fish, some more covered than others. Patches were ~~not~~ most numerous on either side of the body, the fins, particularly the dorsal, anal,

Specimen Submitted: Live Animal ☐ Dead Animal ☒ Tissues ☐

Preservation: Fresh ☒ Frozen ☐ Fixed ☐

Condition of Specimen When Received at Lab:

Biospy Data Exact Location	Size	Duration	Encapsulated	Lymph node involvement
	42 cm		YES ☐ NO ☐	YES ☐ NO ☐

Autopsy Data Natural Death ☐	Mode of Euthanasia	Time and Date of Death	Time and Date of Autopsy

Tissues Submitted:

Findings:
and caudal, and on the lips. Fish were taken from 1 meter to 5 meters deep on 3" plastic tail jig lures and 4" plastic worms -- from weedy areas (submergents) to rocky shorelines - also adjacent to sunken barges. Water temp. - 9°C. Clarity <1 meter. Tidal amplitude approx. 1 meter. Weather conditions overcast, 38°, northerly breeze.

There are round to rectangular pigmented (black) areas above lateral line and on dorsal fin. Areas are 4 x 2 to 0.5 x 1 cm and do not extend into dermis. They number 8. The anal fin also has such an area. Mesentary was adhering to the peritoneum.

SEE OVER

Diagnoses: Peritonitis, Granulomatous, Verminous, Melanosis

Classification: P8 2a U3C

Tissues saved ☐
Photographs ☐
Radiographs ☐

Pathologist:

Date Prepared: 12-14-84

Fee:

A. Myocardium - NSL. Hindgut - NSL. Pancreas - NSL. Mesentery - focal granulomas with generalized histiocytic response.
B. Gill - NSL. Stomach - NSL. Liver - +2 MA, NSL.
C. Kidney - myoxosporidiosis. Brain - NSL.
D. Skin and Scale - shattered in cutting however theee is an excessive pigment deposition ón èpidermis. The malpigian layers above the stratum germanitivum are infiltrated by dendritic melanocytes. However the pigment in the dermis just below the basement membrane is absent.

E. Fin in cross section - see above (D) plus one notes an influx of coarse eosinophilic cells and a disorganization of malpigean cells. The number of mucus cells is decreased.

G. Lateral Line - NSL. Nares - NSL.

Very interesting condition that might well be the result of pollution. This bears further examination - will send to Smithsonian for collection.
