# Supplementary figures and images for: Novel adomaviruses associated with blotchy bass syndrome in black basses (Micropterus spp.)
Source: PLoS One. 2025 Dec 17;20(12):e0326402. doi: 10.1371/journal.pone.0326402 (PMC12711042; doi:10.1371/journal.pone.0326402)

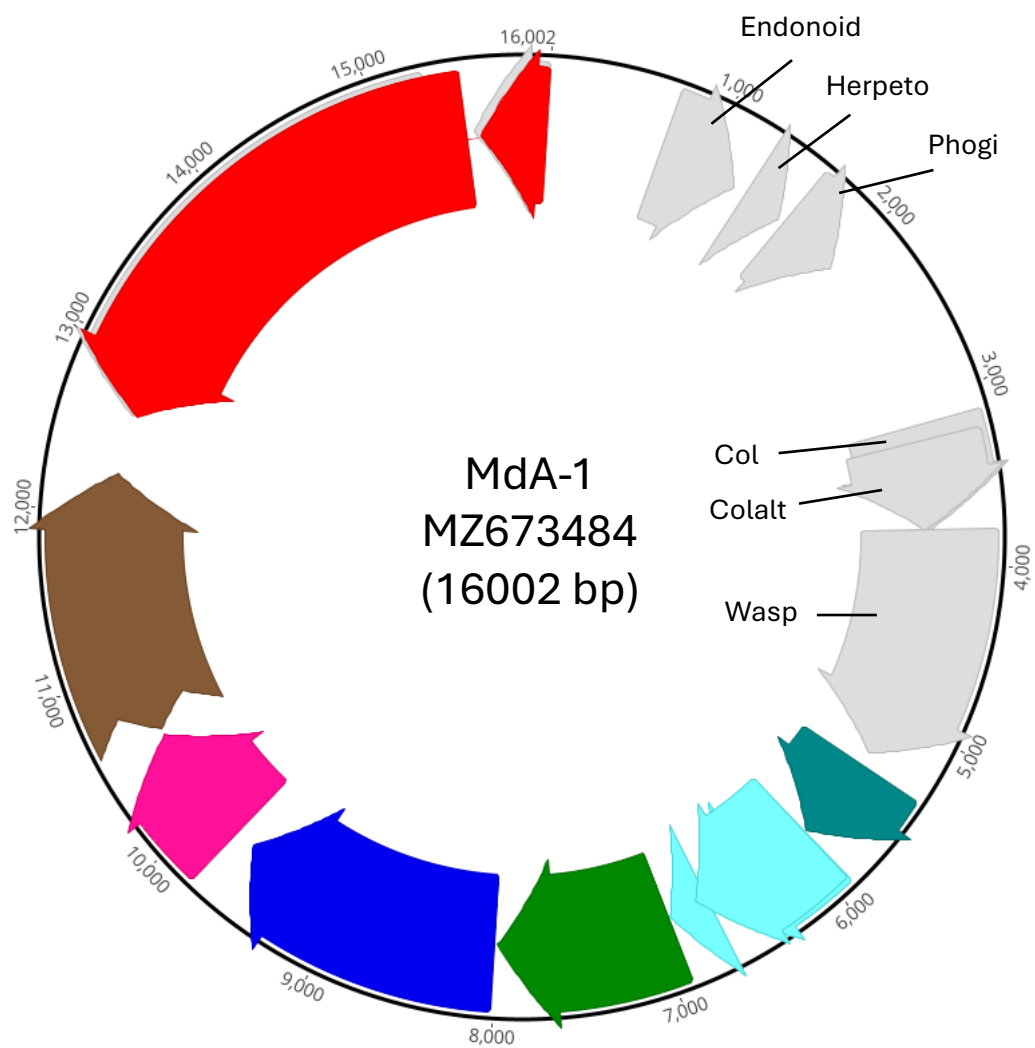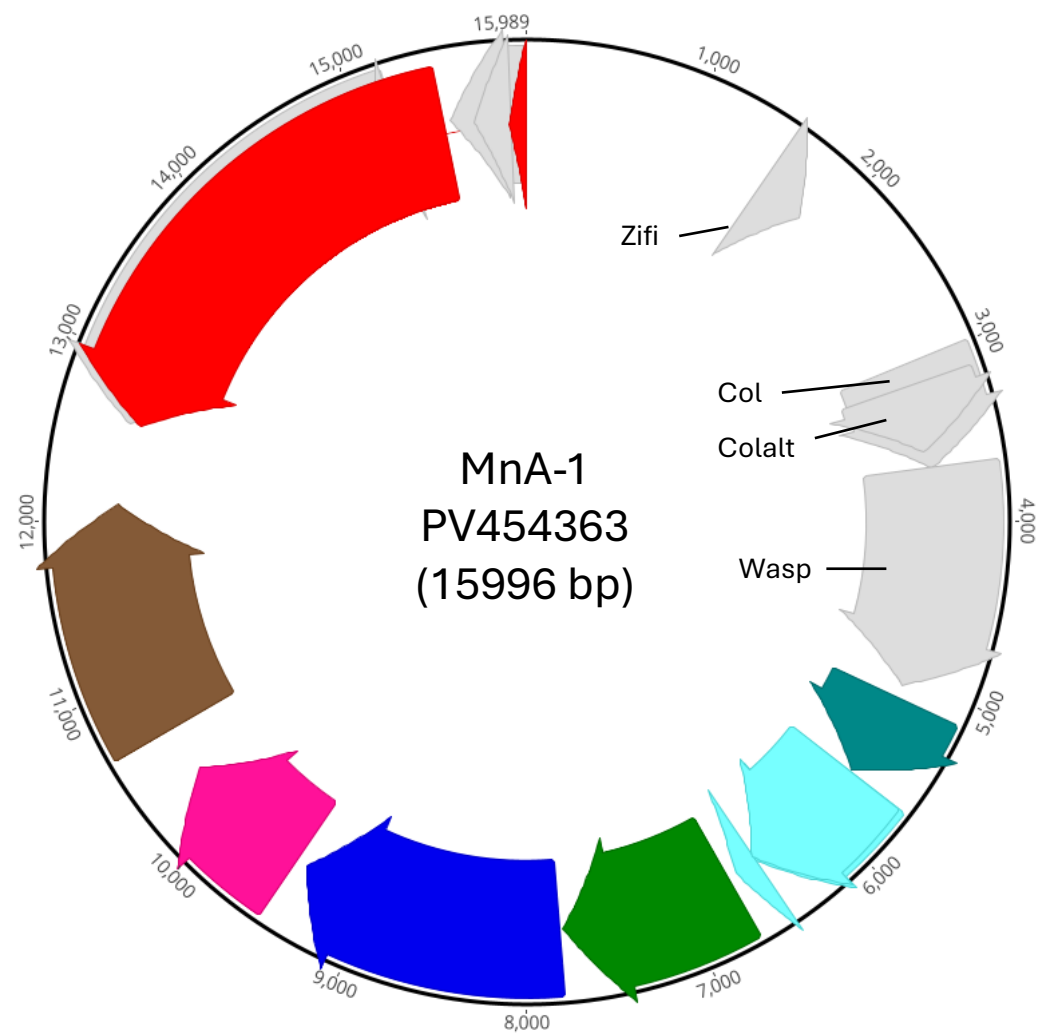

■ Cah 
 ■ Penton 
 ■ Macc 
 ■ Hexon 
 ■ Adenain 
 ■ Prim 
 ■ RepE1

Supplement: S2 Fig — Genome sizes and GenBank accession numbers are indicated. Core adomavirus ORFs are color-coded and specified. Other ORFs are gray. (PDF) [file pone.0326402.s002.pdf]

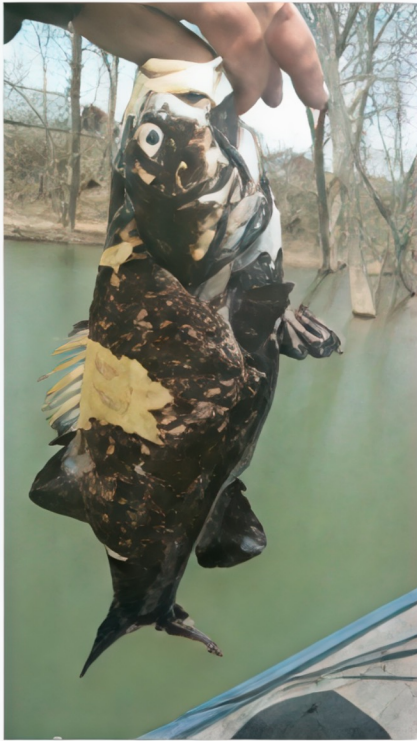

Melanistic lesion

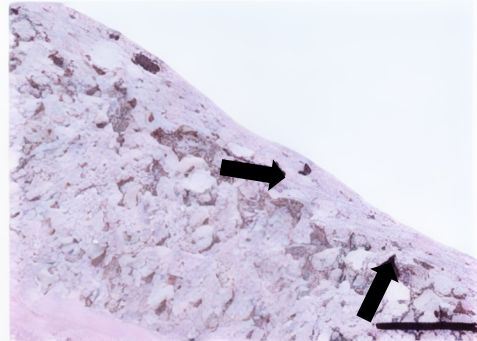

Normal skin

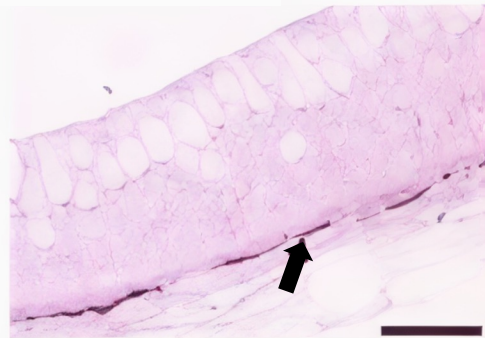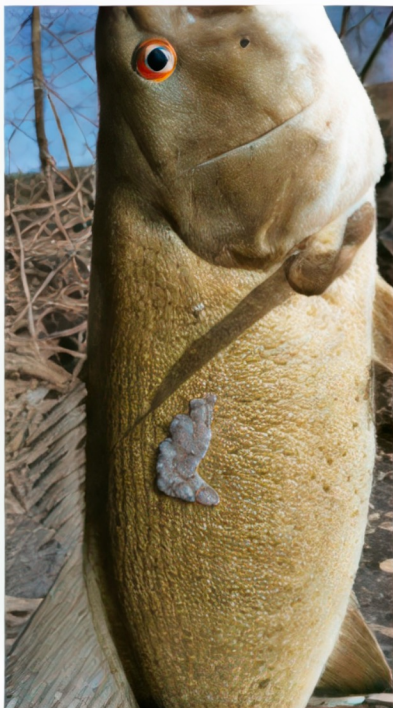

Mucoid lesion

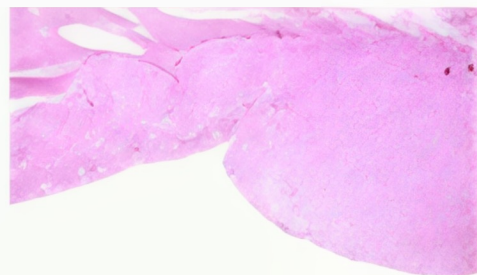

Supplement: S3 Fig — (PDF) [file pone.0326402.s003.pdf]

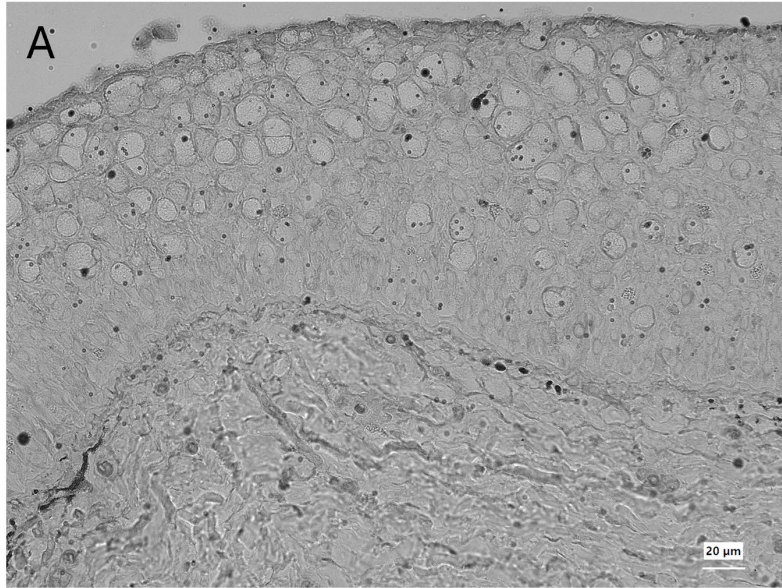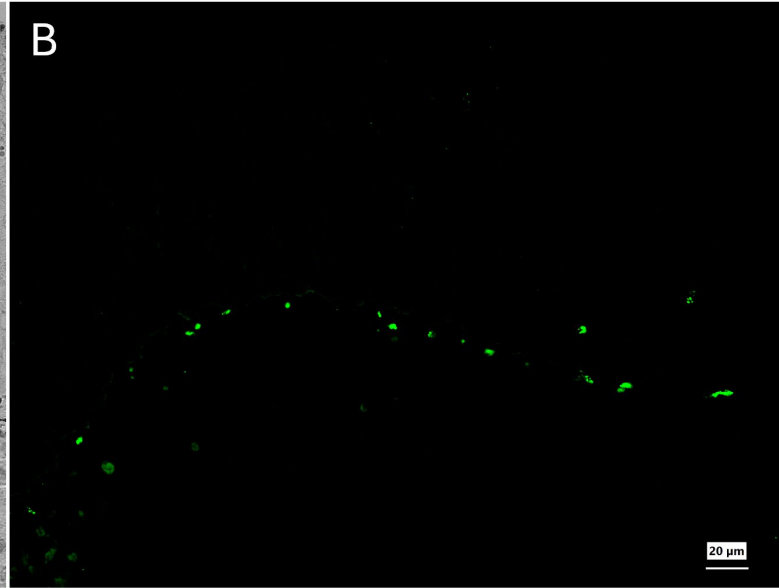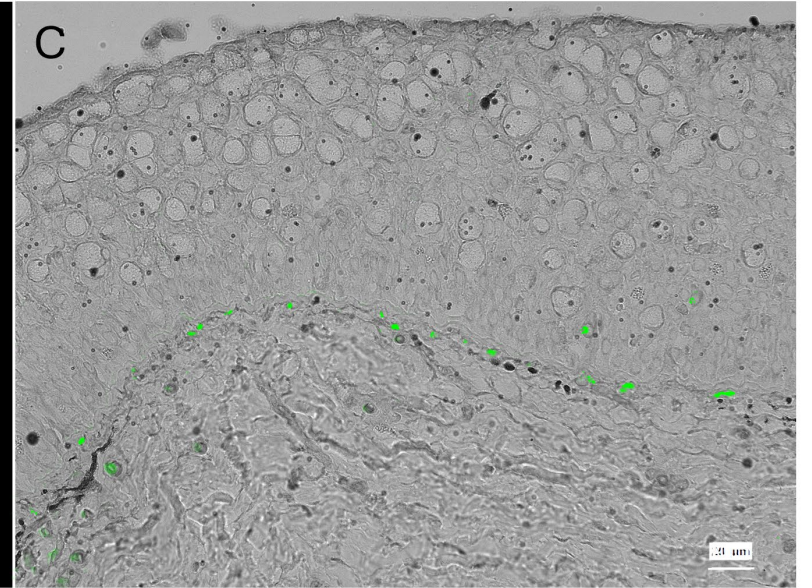

Supplement: S5 Fig — The Adenain transcript of MnA-1 was targeted. Cells positive for adomavirus nucleic acids (green) are observed in the epidermis, but most are observed in the basement membrane. Panels: A; brightfield, B; darkfield and C; composite overlay. (PDF) [file pone.0326402.s005.pdf]

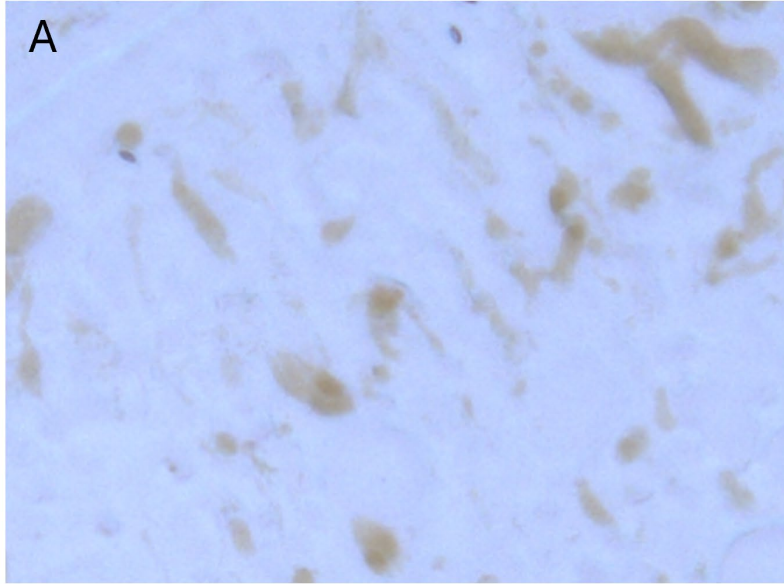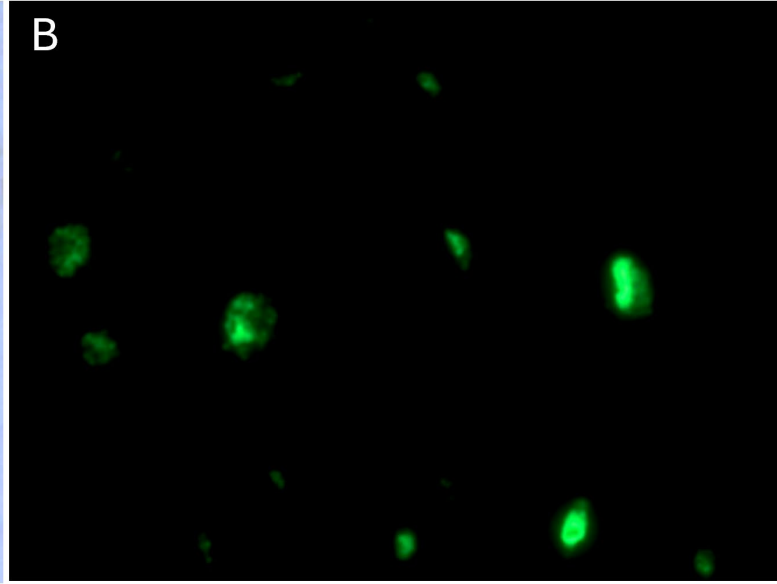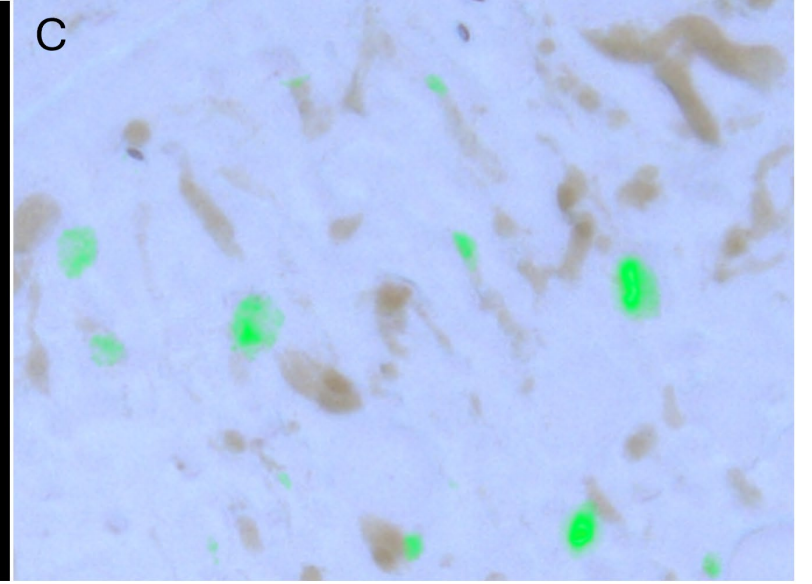

Supplement: S6 Fig — The Adenain transcript of MdA-1 was targeted. Cells positive for adomavirus nucleic acids (green) are restricted to the epidermis. While viral nucleic acids were sometimes observed in melanocytes, they were more commonly observed in non-pigmented, epithelial cells of the epidermis. (PDF) [file pone.0326402.s006.pdf]

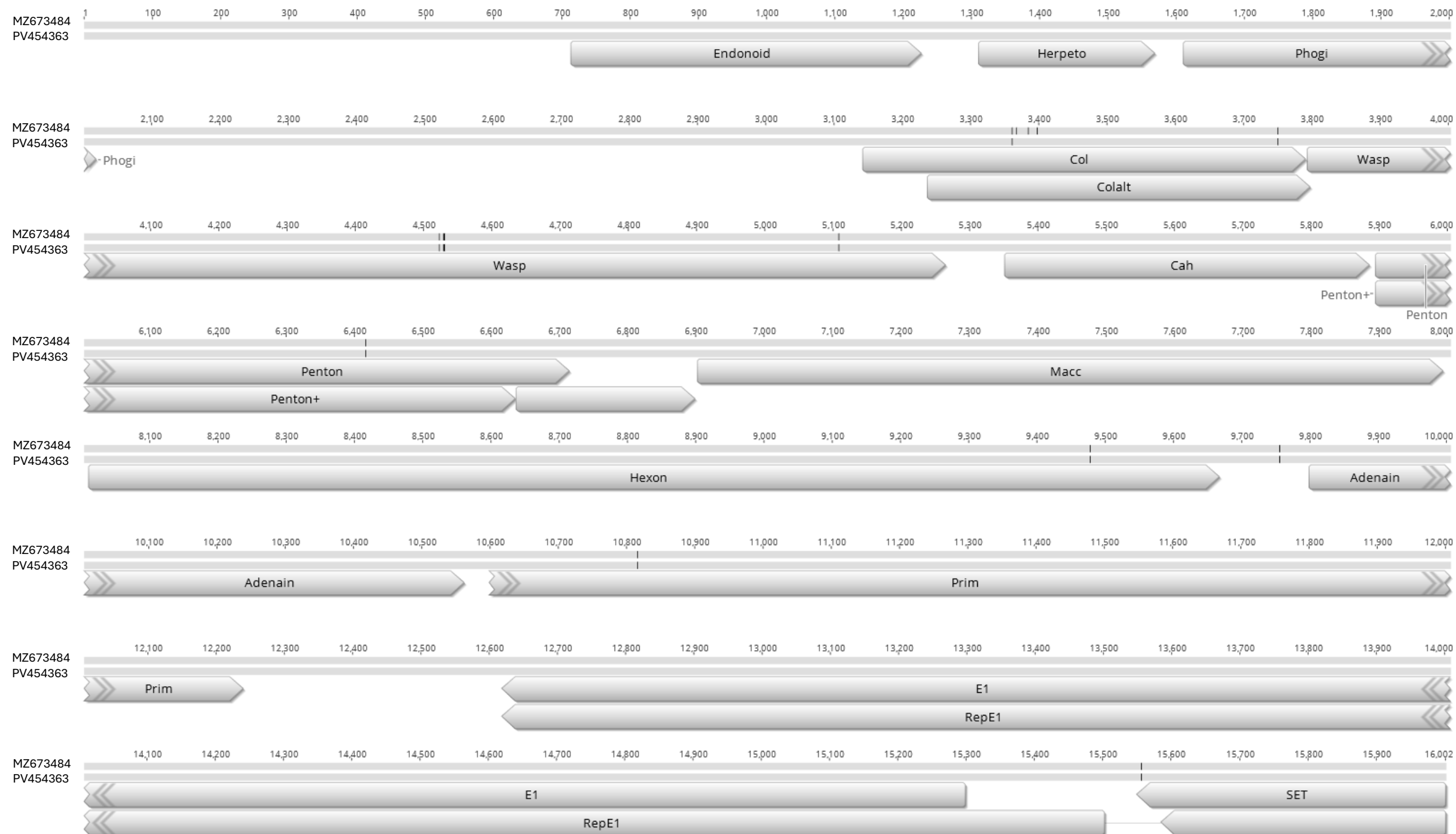

Supplement: S7 Fig — Genomes represent MdA-1 isolated from smallmouth bass inhabiting different rivers in Pennsylvania collected during different years. SNPs are indicated with vertical lines. Open reading frames are identified. (PDF) [file pone.0326402.s007.pdf]
