# Supplementary material for: Novel adomaviruses associated with blotchy bass syndrome in black basses (Micropterus spp.)
Source: PLoS One. 2025 Dec 17;20(12):e0326402. doi: 10.1371/journal.pone.0326402 (PMC12711042; doi:10.1371/journal.pone.0326402)
Supplement: S4 Fig — Pairwise identity of homologous MdA-1 and MnA-1 proteins are indicated. (PDF) [file pone.0326402.s004.pdf]

Col

Pairwise Identity = 63.4%

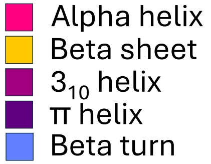

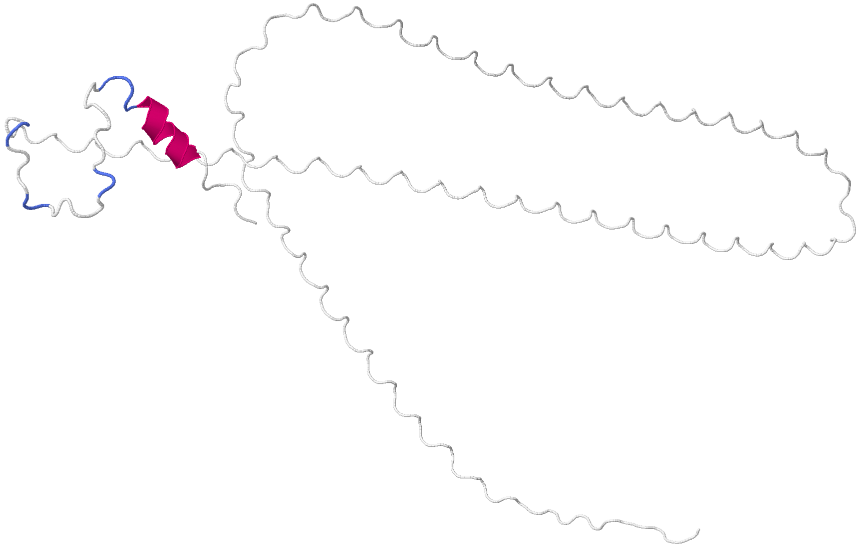

MdA-1
(UFQ21626)

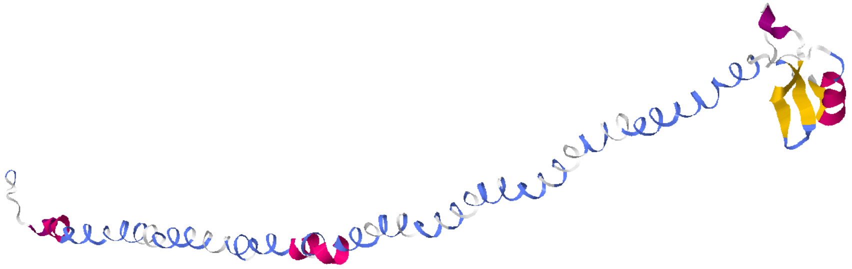

MnA-1
(XQZ12361)

Colalt

Pairwise Identity = 41.0%

Alpha helix
Beta sheet
3_{10} helix
π helix
Beta turn

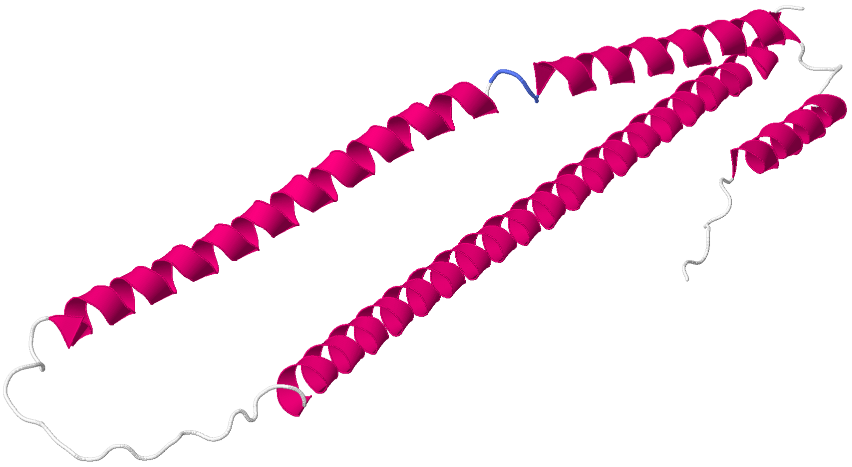

MdA-1
(UFQ21627)

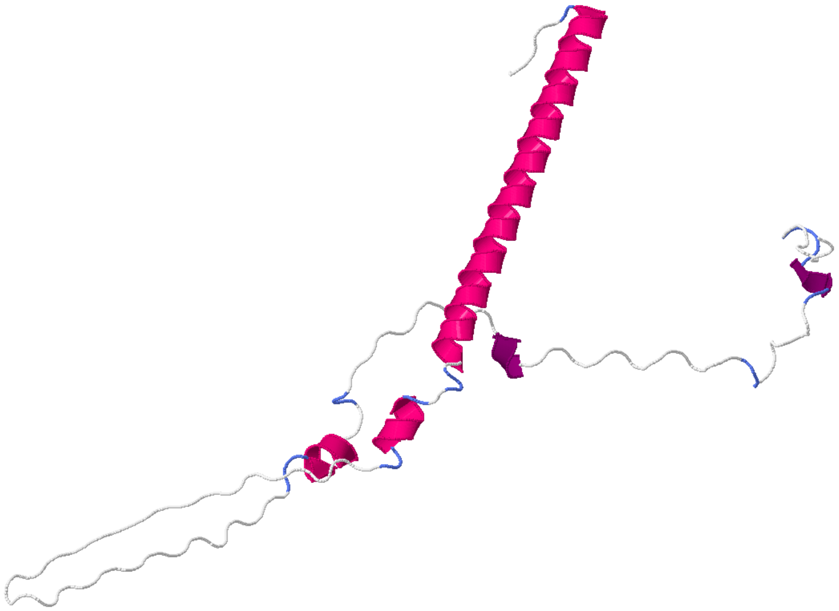

MnA-1
XQZ12362

Wasp

Pairwise Identity = 51.7%

- Alpha helix
- Beta sheet
- 3_{10} helix
- π helix
- Beta turn

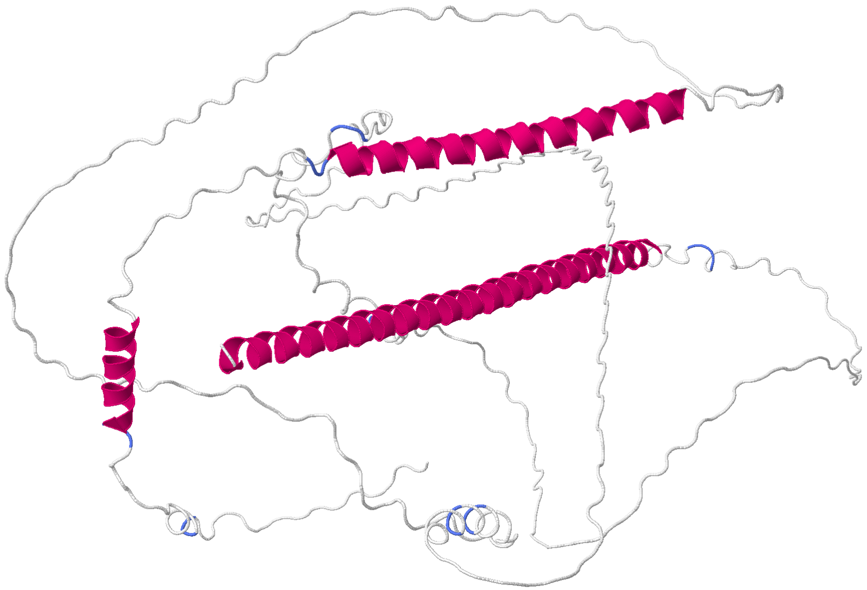

MdA-1
(UFQ21628)

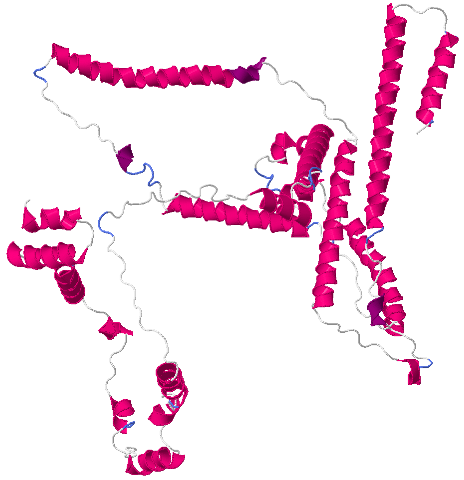

MnA-1
(XQZ12363)

Cah

Pairwise Identity = 82.5%

- Alpha helix
- Beta sheet
- 3_{10} helix
- π helix
- Beta turn

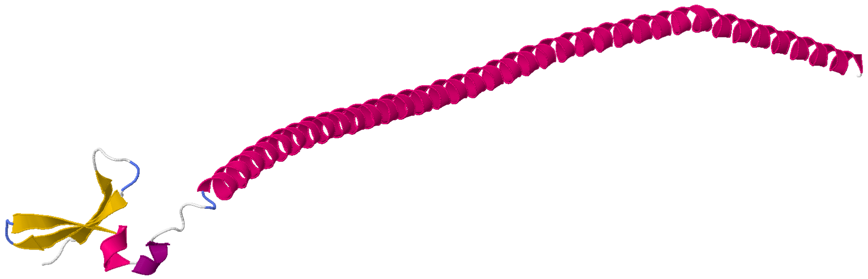

MdA-1
(UFQ21629)

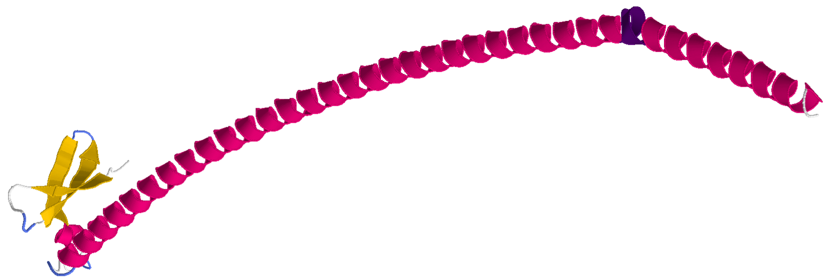

MnA-1
(XQZ12364)

Macc

Pairwise Identity = 74.0%

- Alpha helix
- Beta sheet
- 3_{10} helix
- π helix
- Beta turn

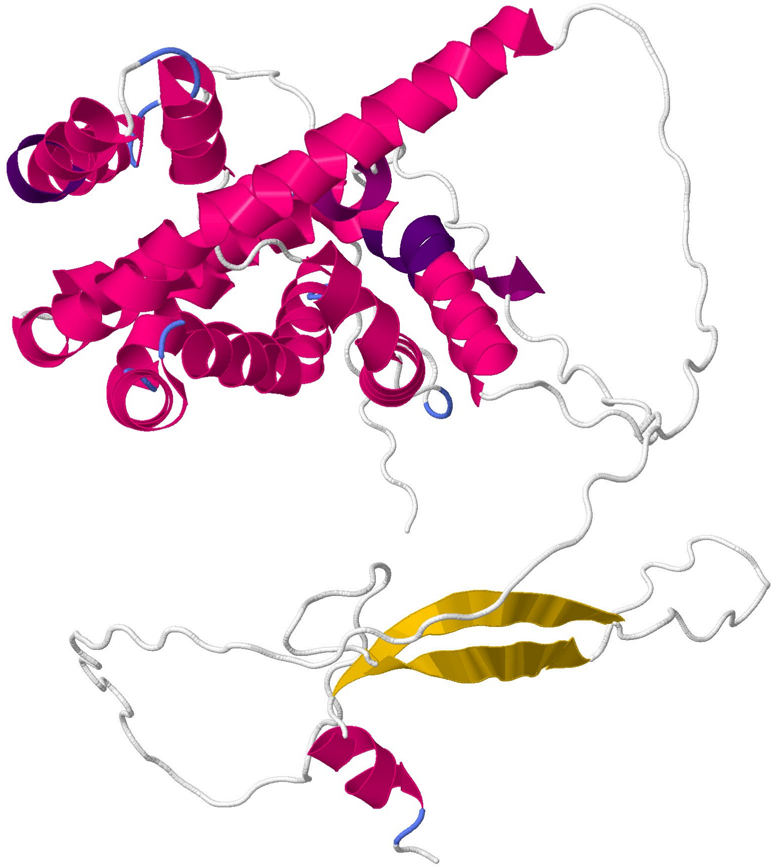

MdA-1
(UFQ21631)

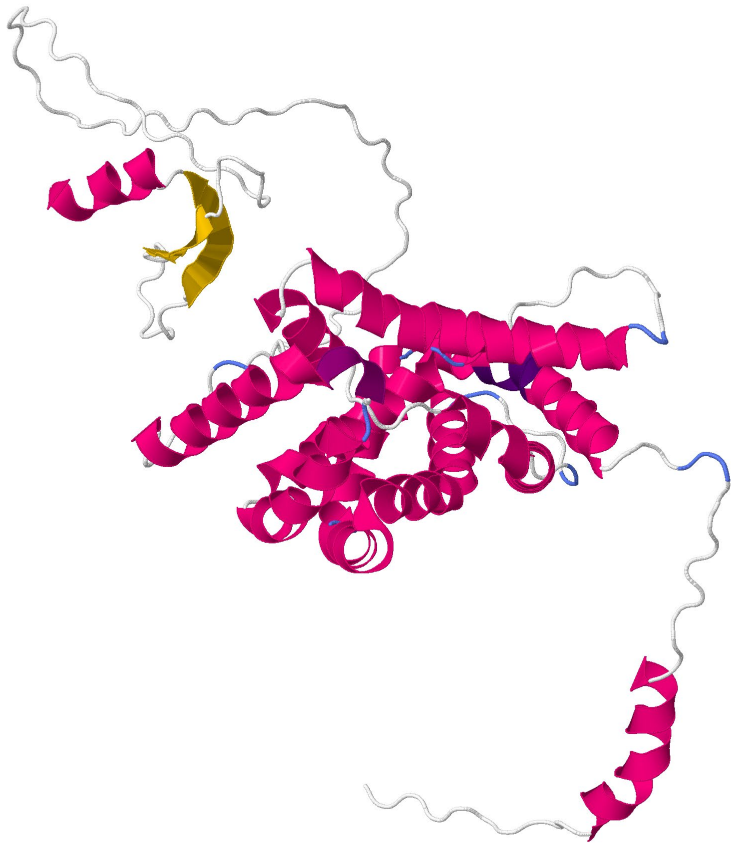

MnA-1
(XQZ12367)

Penton

Pairwise Identity = 86.8%

Alpha helix
Beta sheet
3_{10} helix
π helix
Beta turn

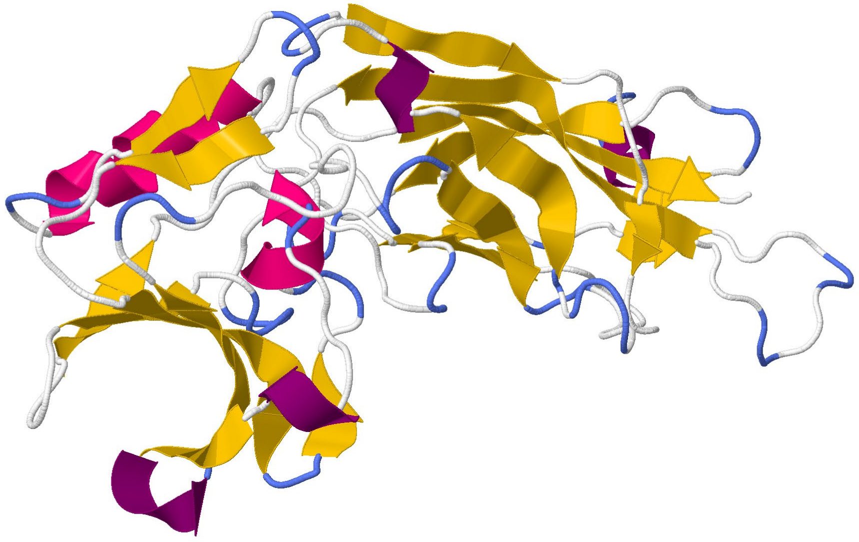

MdA-1
(UFQ21630)

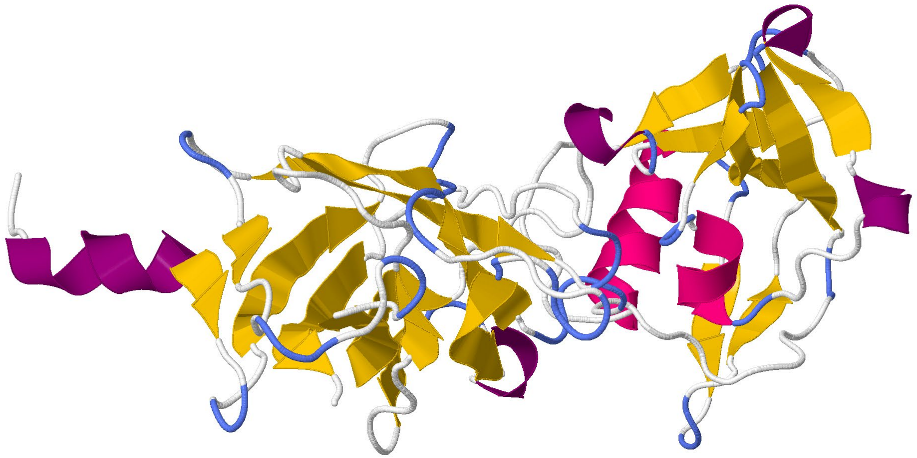

MnA-1
(XQZ12365)

Penton+

Pairwise Identity = 83.4%

- Alpha helix
- Beta sheet
- 3_{10} helix
- π helix
- Beta turn

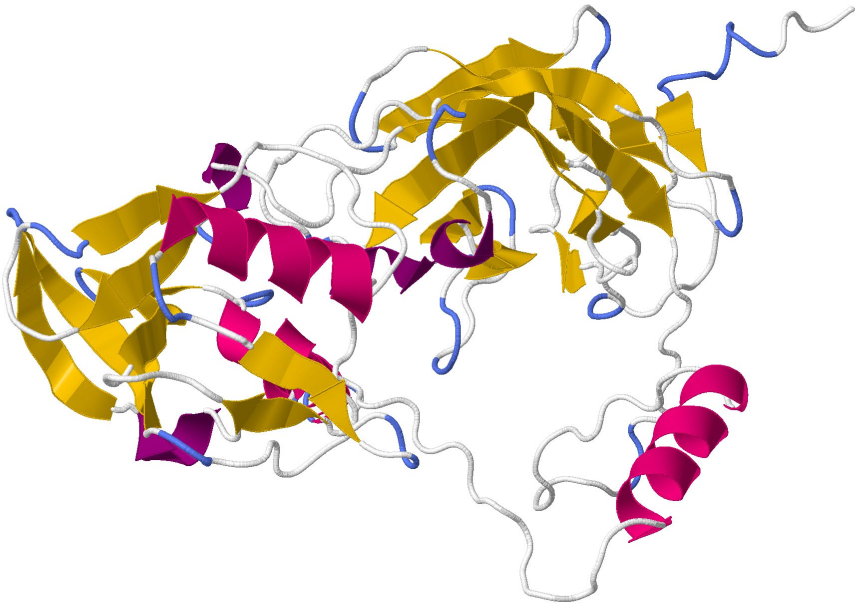

MdA-1
(XQU54337)

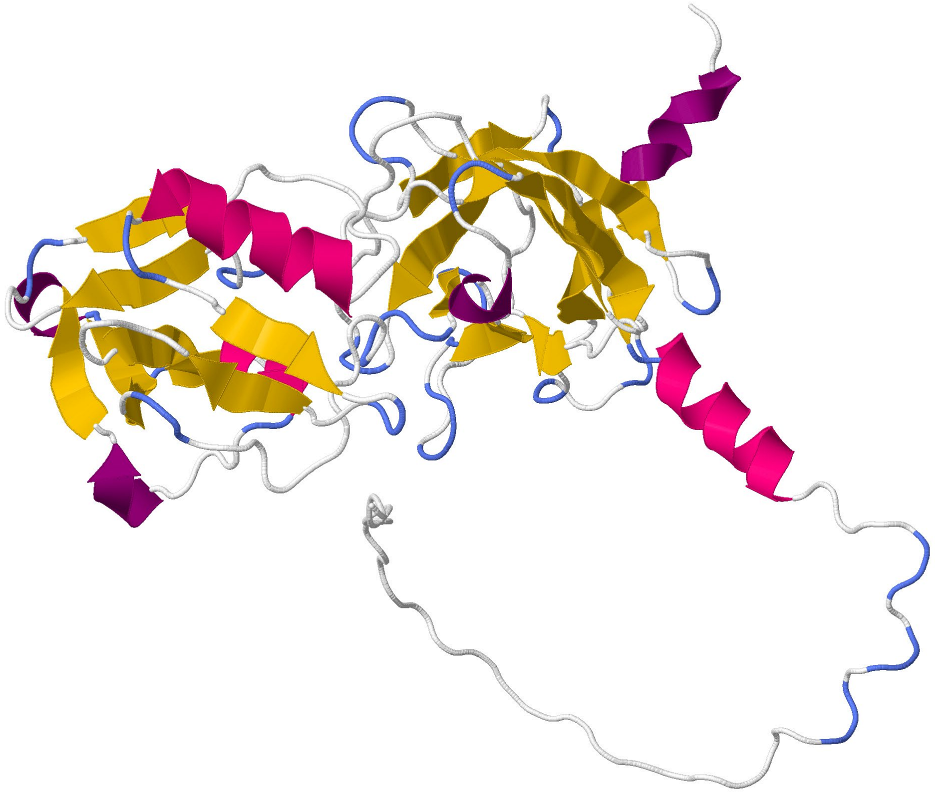

MnA-1
(XQZ12366)

Hexon

Pairwise Identity = 92.2%

- Alpha helix
- Beta sheet
- 3_{10} helix
- π helix
- Beta turn

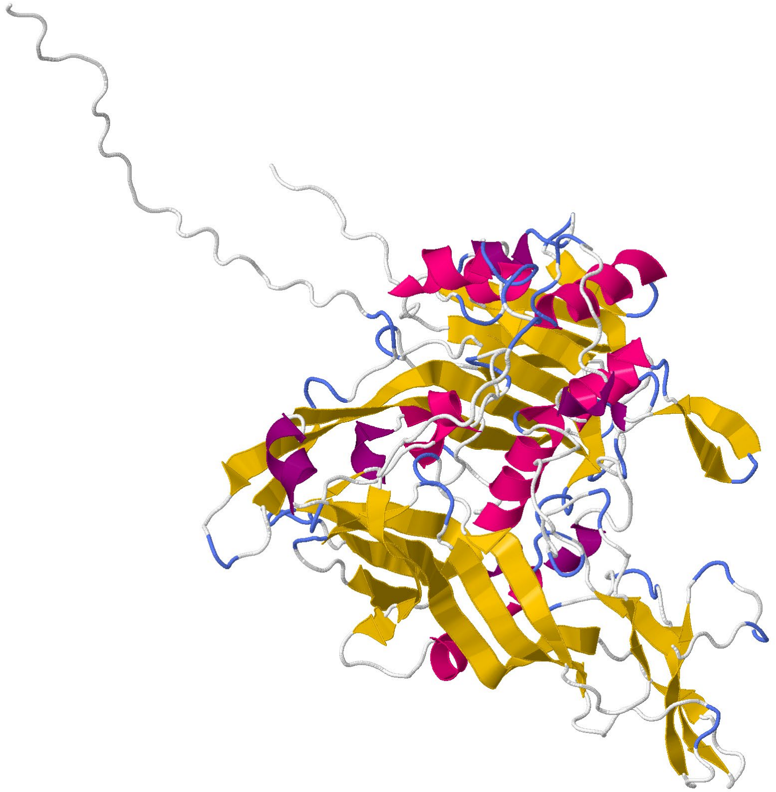

MdA-1
(UFQ21632)

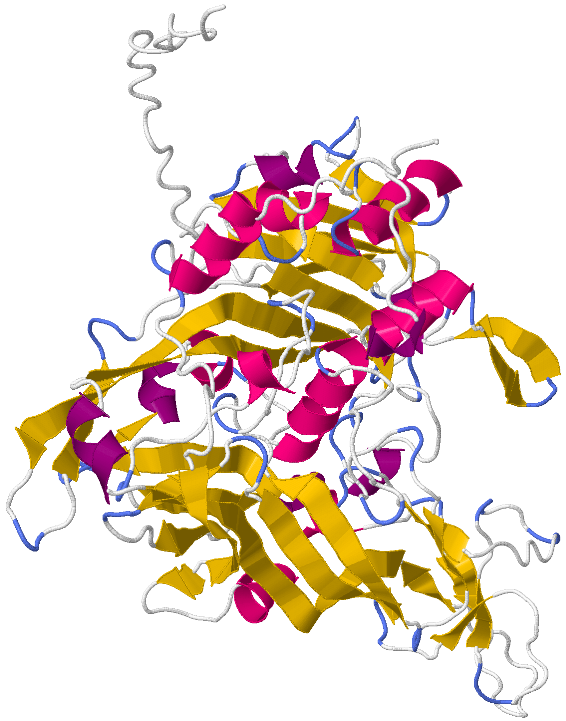

MnA-1
(XQZ1236)

Adenain

Pairwise Identity = 72.4%

- Alpha helix
- Beta sheet
- 3_{10} helix
- π helix
- Beta turn

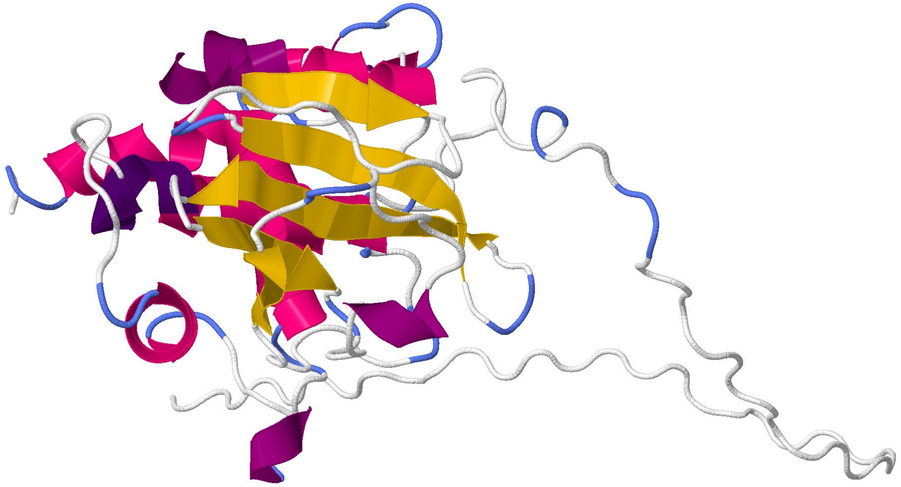

MdA-1
(UFQ21633)

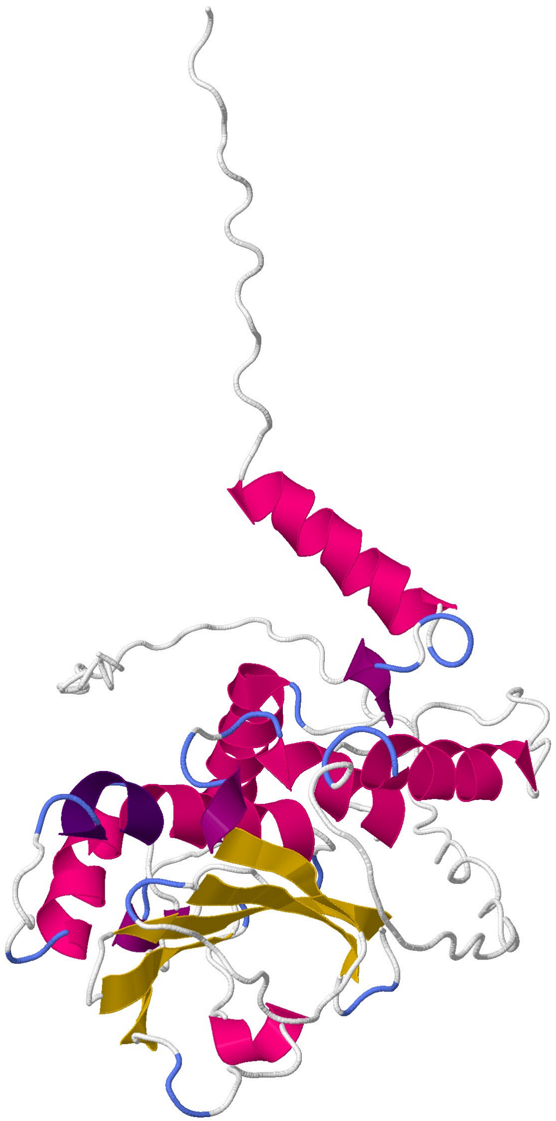

MnA-1
(XQZ12369)

Prim

Pairwise Identity = 76.9%

- Alpha helix
- Beta sheet
- 3_{10} helix
- π helix
- Beta turn

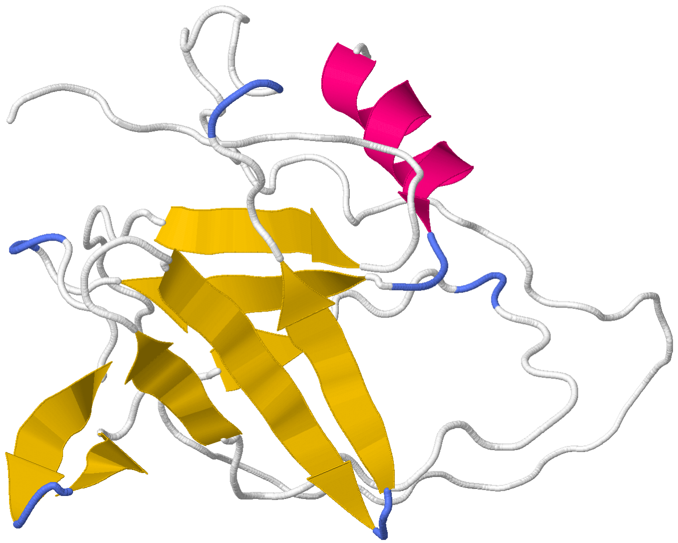

MdA-1
(UFQ21634)

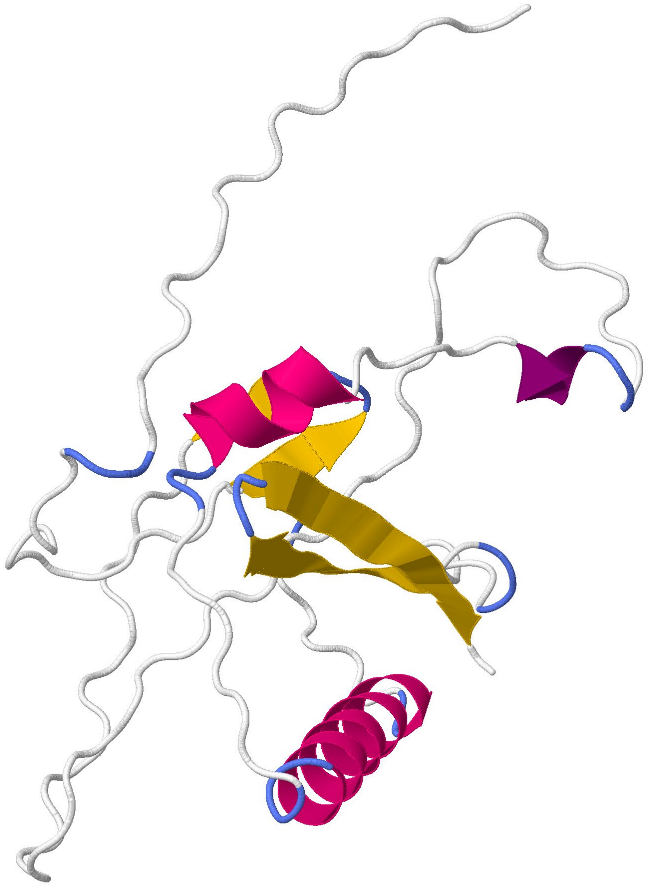

MnA-1
(XQZ12370)

RepE1

Pairwise Identity = 78.9%

Alpha helix
Beta sheet
3_{10} helix
π helix
Beta turn

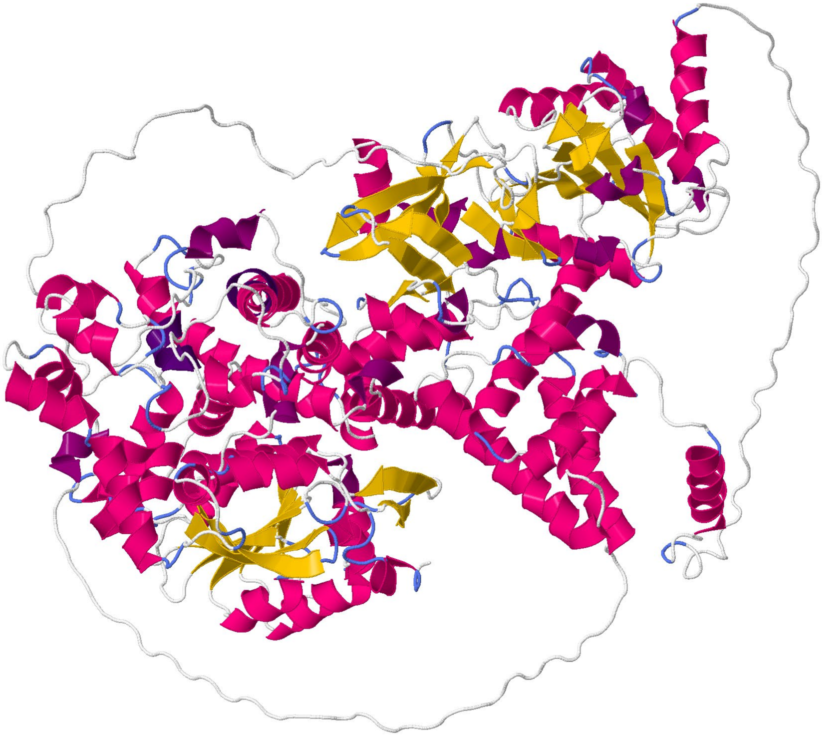

MdA-1
(UFQ21635)

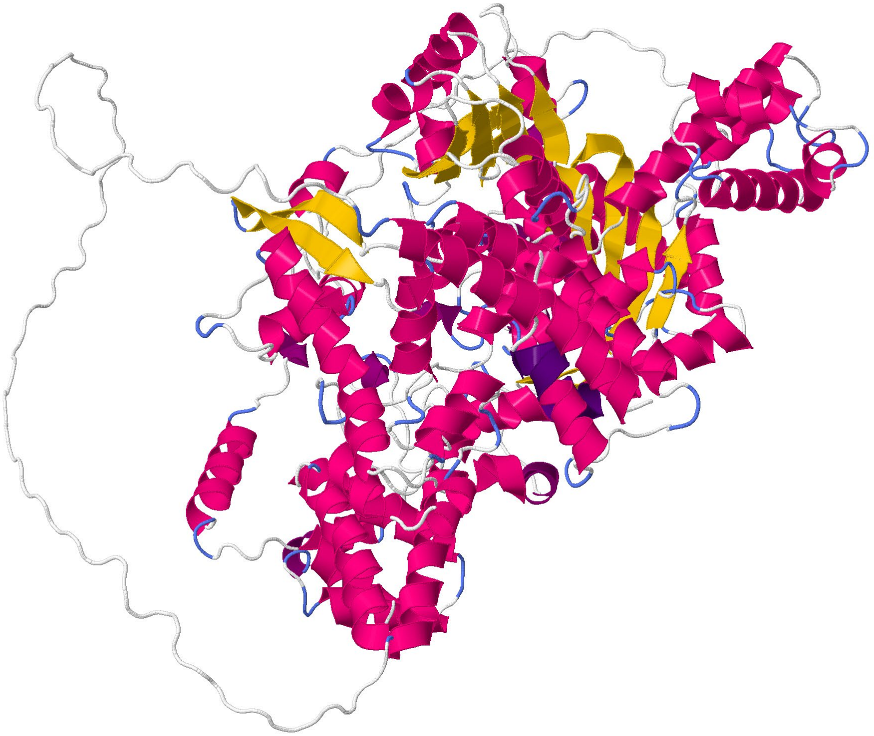

MnA-1
(XQZ12371)

SET

Pairwise Identity = 62.0%

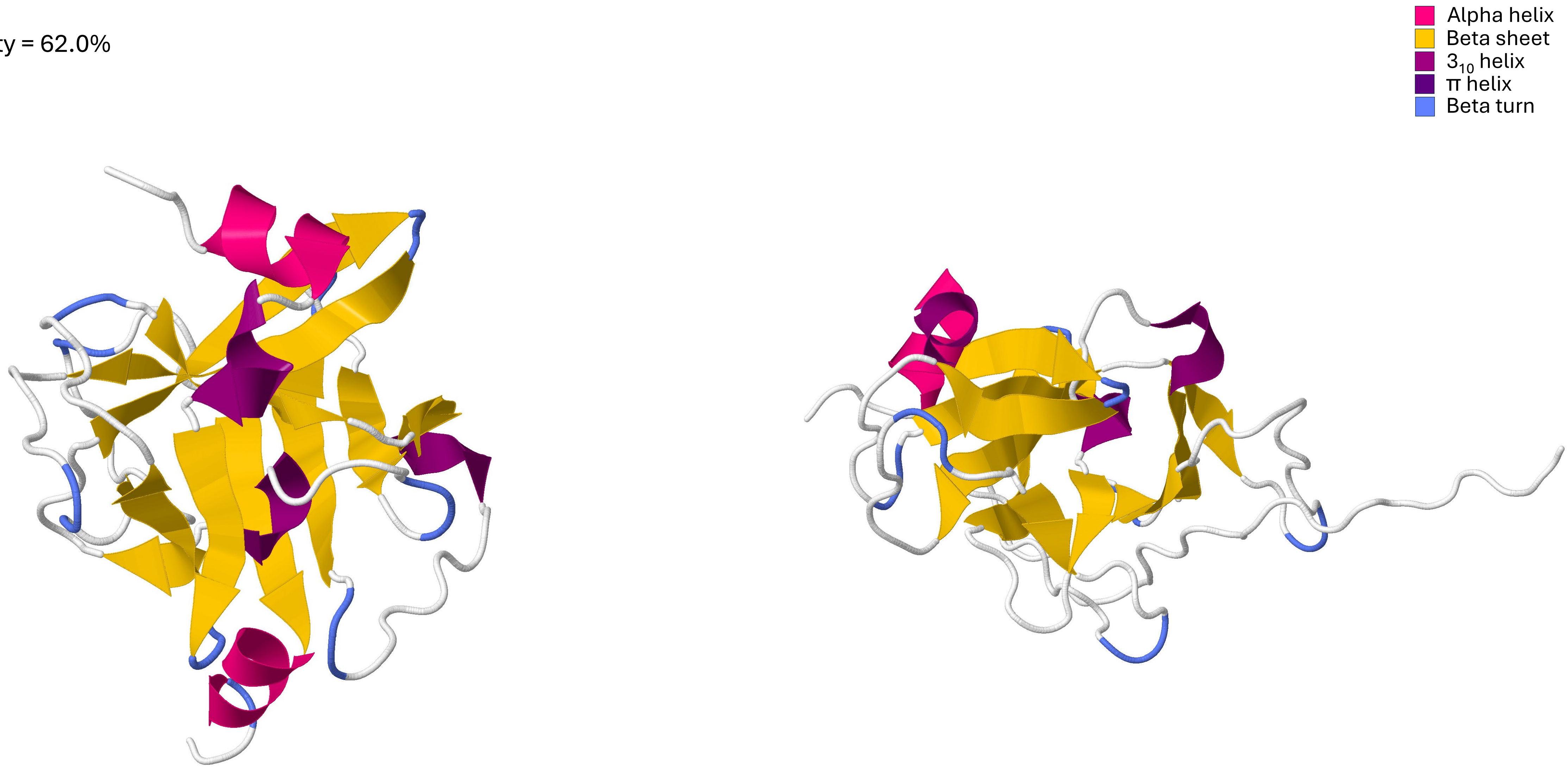

MdA-1
(UFQ21636)

MnA-1
(XQZ12374)

Zifi

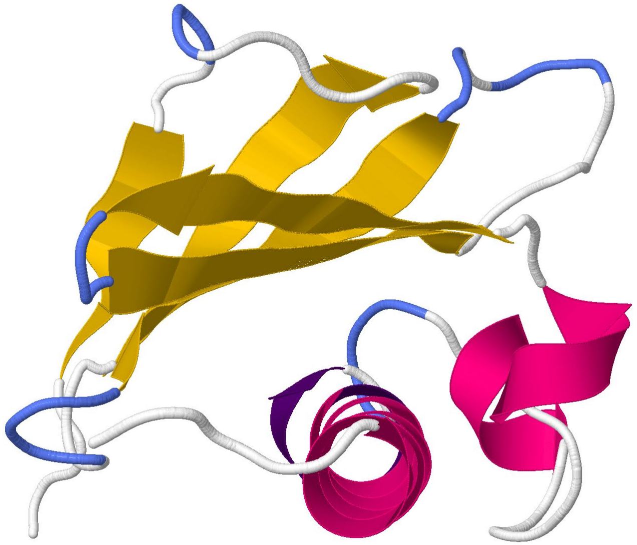

MnA-1
(XQZ12360)

Alpha helix
Beta sheet
3_{10} helix
π helix
Beta turn

Endonoid

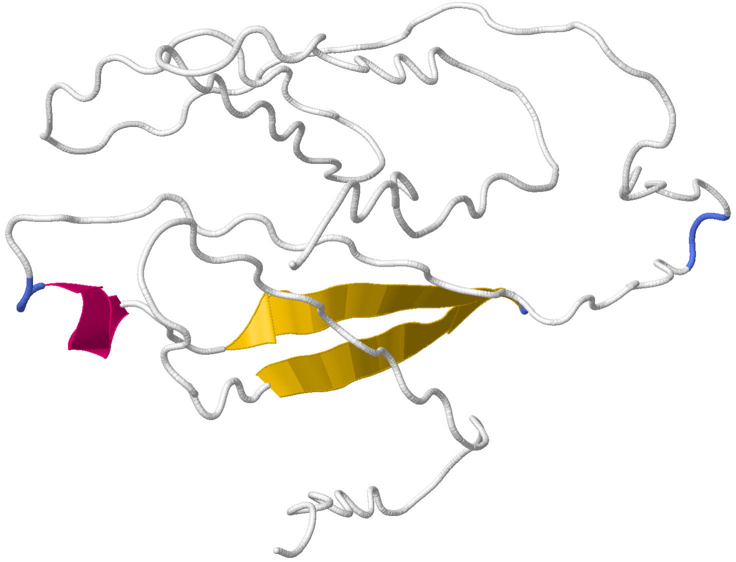

MdA-1
(UFQ21624)

Herpeto

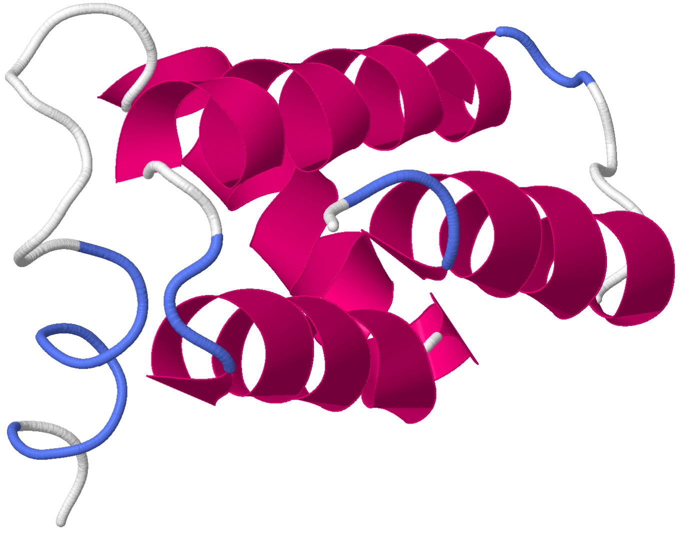

MdA-1
(XQU54336)

Phogi

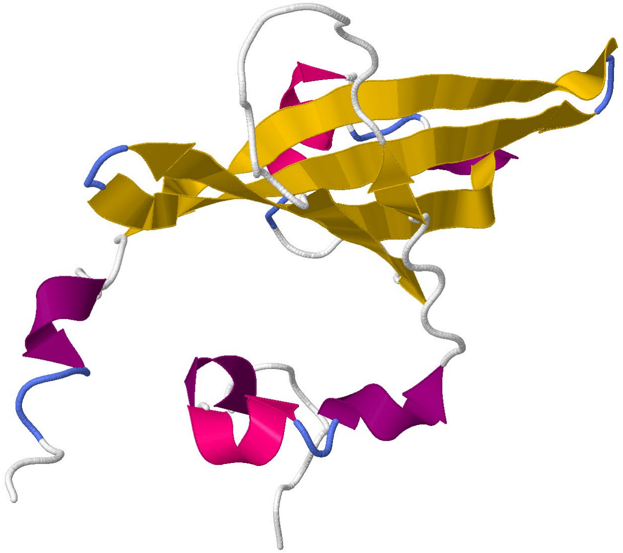

MdA-1
(UFQ21625)
