# Supplementary material for: Novel adomaviruses associated with blotchy bass syndrome in black basses (Micropterus spp.)
Source: PLoS One. 2025 Dec 17;20(12):e0326402. doi: 10.1371/journal.pone.0326402 (PMC12711042; doi:10.1371/journal.pone.0326402)
Supplement: S8 Fig — Adomavirses with cichlid hosts are most similar to MdA-1 and MnA-1. Core adomavirus ORFs are colored in non-gray. Identity graph depicts similarity/dissimilarity across the genomes (sliding window = 1). (PDF) [file pone.0326402.s008.pdf]

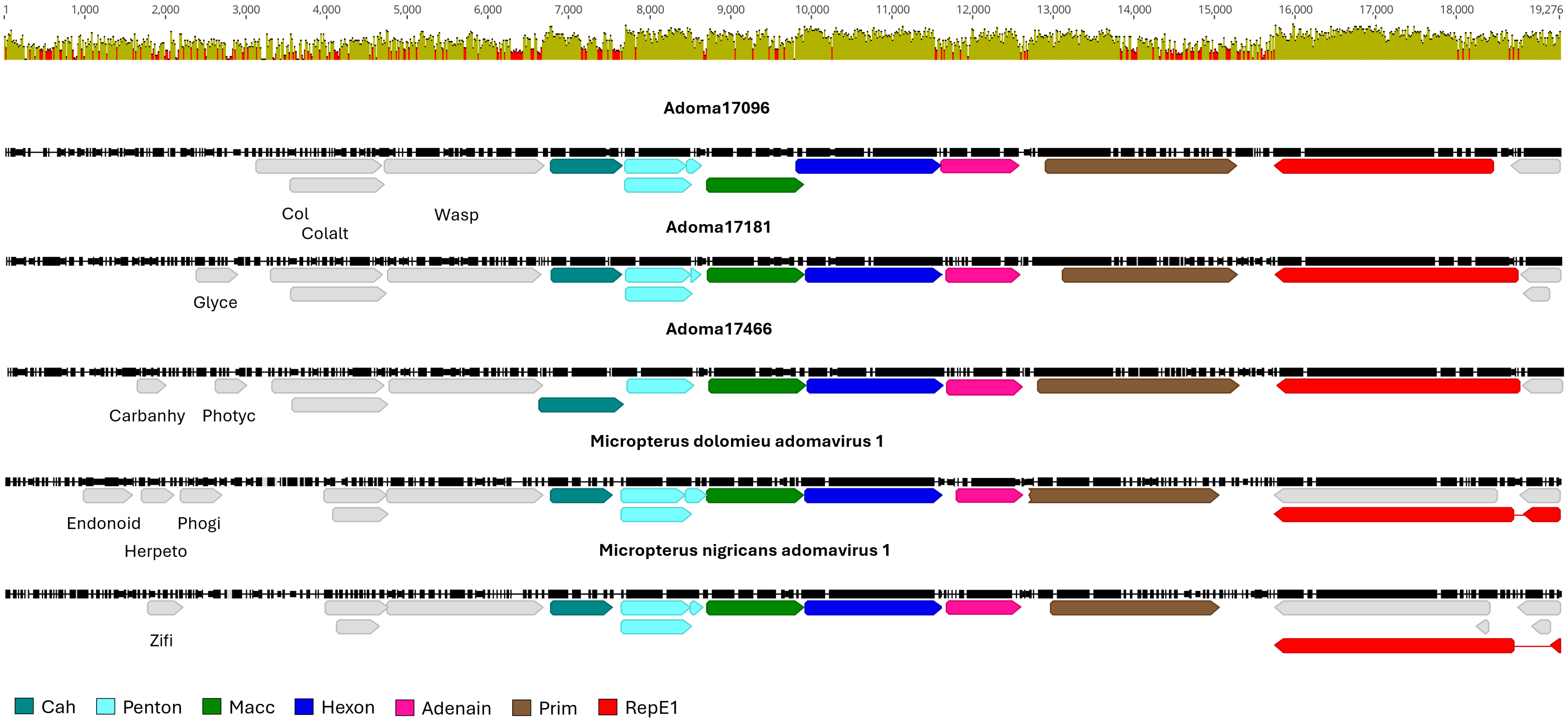
1
1,000
2,000
3,000
4,000
5,000
6,000
7,000
8,000
9,000
10,000
11,000
12,000
13,000
14,000
15,000
16,000
17,000
18,000
19,276
Adoma17096
Col
Colalt
Wasp
Adoma17181
Glyce
Adoma17466
Carbanhy
Photyc
Micropterus dolomieu adomavirus 1
Endonoid
Herpeto
Phogi
Micropterus nigricans adomavirus 1
Zifi
Cah
Penton
Macc
Hexon
Adenain
Prim
RepE1
